# Supplementary material for: Identification and functional analysis of non-coding regulatory small RNA FenSr3 in Bacillus amyloliquefaciens LPB-18
Source: PeerJ. 2023 May 15;11:e15236. doi: 10.7717/peerj.15236 (PMC10194069; doi:10.7717/peerj.15236)
Supplement: Supplemental Information 4 [file peerj-11-15236-s004.zip › KO/CK-vs-T1_map/map04721.html]

KEGG PATHWAY: Synaptic vesicle cycle - Reference pathway


|  |  |
| --- | --- |
| **Synaptic vesicle cycle - Reference pathway** |  |

[
Pathway menu
| Organism menu
| Pathway entry
| Show description
| User data mapping
]

Communication between neurons is mediated by the release of neurotransmitter from synaptic vesicles (SVs). At the nerve terminal, SVs cycle through repetitive episodes of exocytosis and endocytosis. SVs are filled with neurotransmitters by active transport. The loaded SVs are then docked at a specialized region of the presynaptic plasma membrane known as the active zone, where they undergo a priming reaction. Upon arrival of an action potential, Ca2+ enters through voltage-gated channels and neurotransmitter is released by exocytosis, usually in less than a millisecond. After fusion, the vesicle is retrieved by endocytosis and reloaded for another round of exocytosis.

|  |  |  |
| --- | --- | --- |
| Reference pathway | 184% 150% 122% 100% 82% 67% 55% | 图片下载 |
